# Variability Across Caregiver and Performance-Based Measures of Executive Functioning in an Acute Pediatric Neurocritical Care Population

**DOI:** 10.1089/neur.2022.0083

**Published:** 2023-03-01

**Authors:** Kera R. Larson, Lauren A. Demers, Emily Z. Holding, Cydni N. Williams, Trevor A. Hall

**Affiliations:** ^1^ Division of Pediatric Psychology, Department of Pediatrics, Oregon Health & Science University, Portland, Oregon, USA.; ^2^ Division of Pediatric Critical Care, Department of Pediatrics, Oregon Health & Science University, Portland, Oregon, USA.; ^3^ Pediatric Critical Care and Neurotrauma Recovery Program, Oregon Health & Science University, Portland, Oregon, USA.; ^4^ Developmental Medical Center, Boston Children's Hospital, Boston, Massachusetts, USA.

**Keywords:** executive functioning, neurocritical care, pediatric, traumatic brain injury

## Abstract

Youth admitted to the pediatric intensive care unit (PICU) for traumatic brain injury (TBI) commonly struggle with long-term residual effects in the domains of physical, cognitive, emotional, and psychosocial/family functioning. In the cognitive domain, executive functioning (EF) deficits are often observed. The Behavior Rating Inventory of Executive Functioning, Second Edition (BRIEF-2) is a parent/caregiver-completed measure that is regularly utilized to assess caregivers' perspectives of daily EF abilities. Using parent/caregiver-completed measures like the BRIEF-2 in isolation as outcome measures for capturing symptom presence and severity might be problematic given that caregiver ratings are vulnerable to influence from external factors. As such, this study aimed to investigate the association between the BRIEF-2 and performance-based measures of EF in youth during the acute recovery period post-PICU admission for TBI. A secondary aim was to explore associations among potential confounding factors, including family-level distress, injury severity, and the impact of pre-existing neurodevelopmental conditions. Participants included 65 youths, 8–19 years of age, admitted to the PICU for TBI, who survived hospital discharge and were referred for follow-up care. Non-significant correlations were found between BRIEF-2 outcomes and performance-based measures of EF. Measures of injury severity were strongly correlated with scores from performance-based EF measures, but not BRIEF-2. Parent/caregiver-reported measures of their own health-related quality of life were related to caregiver responses on the BRIEF-2. Results demonstrate the differences captured by performance-based versus caregiver-report measures of EF, and also highlight the importance of considering other morbidities related to PICU admission.

## Introduction

Traumatic brain injury (TBI) continues to be the leading cause of morbidity and mortality in children and adolescents, with neurological injury accounting for >20% of youth admitted to the pediatric intensive care unit (PICU).^1–4^ Though the efficacy of PICU-specific intervention has increased survival rates, many children and families face worsening functional outcomes after discharge.^5^ PICU survivors often experience long-term sequelae or post-intensive care syndrome, which includes physical, cognitive, emotional, and psychosocial impairments.^1,6,7^

Of the cognitive domains affected by TBI, executive functioning (EF) is often the most severely impacted.^8,9^ EF encompasses the skills necessary to achieve a given goal by means of regulating thoughts, behaviors, and emotions and includes skills such as problem solving, self-monitoring, task initiation/completion, cognitive flexibility, and behavioral/emotional modulation.^10^ Pronounced weaknesses in this population are observed given that pediatric TBI often affects the neural circuitry underlying EF abilities, including frontal regulatory areas of the brain or diffuse axonal injury.^11–15^

A multi-modal approach is recommended to assess functioning and track TBI recovery post-PICU admission; however, neuropsychological evaluations indexing EF are seldom completed during the acute recovery phase.^1,16,17^ More often, in structured PICU follow-up programs and research protocols, EF is measured using tools such as the Behavior Rating Inventory of Executive Functioning, Second Edition (BRIEF-2), an informant-reported measure designed to assess daily EF.^18–22^ In fact, the BRIEF-2 is recommended by the National Institutes of Health as a common data element for TBI research and is often utilized as a sole measure of EF.^21,23,24^ Clinically, the BRIEF/BRIEF-2 has been widely utilized as an accepted way to assess and track EF deficits and recovery across ages.^25–30^ However, some have proposed that parent/caregiver-reported measures are not ideal as the sole outcome measures for capturing symptom presence and severity. Caregiver ratings tend to be influenced by factors including stress level, skewed recollections of pre-morbid functioning, and response bias.^30–33^

Further, the literature highlights notable discrepancies between informant-reported and performance-based measures of EF. For instance, the BRIEF/BRIEF-2 has been shown to poorly correlate with performance-based measures of EF across numerous pediatric samples unless TBIs were in the moderate to severe range.^34–40^ These findings indicate that parent-reported and performance-based EF measures may capture a different set of constructs (e.g., stress related to PICU admission), particularly when EF challenges are in the more normative or mildly impaired range.^33,34^ More research directly evaluating the connection between family distress and parent-reported EF post-TBI is needed to ensure that EF outcome measures (BRIEF/BRIEF-2) are utilized effectively in TBI populations given both the prominence and longevity of executive dysfunction typically present after the acute recovery phase >10 years post-injury.^28,29,40,41^

Further, earlier studies have been agnostic to time post-injury when studying EF in pediatric TBI. This oversight is, in part, attributable to the rarity of neuropsychological evaluations and rehabilitation programs services available.^29,42^ As such, the research is lacking temporal specificity when it comes to multi-component performance-based assessment of EF, while also accounting for pre-morbid functioning, injury severity, and parent stress. Additionally, pre-existing neurodevelopmental diagnoses are often not reported or addressed, although they are a known risk factor for executive dysfunction beyond injury severity.^26,43–46^

The primary aim of this study was to determine the concordance between parent-reported measures and performance-based measures of EF after neurocritical care during the acute recovery period. A secondary aim was to explore possible confounding factors on EF outcomes, including family-level distress, injury severity, and pre-existing neurodevelopmental conditions. We hypothesized that caregiver-reported measures of EF would not be significantly associated with performance-based measures. Conversely, we hypothesized that parent-reported measures would be associated with caregiver-reported measures of family impact and distress. Additionally, we hypothesized that performance-based measures of EF would relate to injury severity. Finally, we hypothesized that there would be group differences across those with and without pre-existing neurodevelopmental diagnoses.

## Methods

### Participants and procedures

We performed a retrospective analysis of data collected on children 8–19 years of age admitted to the PICU for TBI, who had survived hospital discharge and were referred for follow-up care in the Pediatric Critical Care Neurotrauma Recovery Program (PCCNRP) at Doernbecher Children's Hospital ∼1–3 months (mean [*M* ] = 51.72 days, [*SD*] = 16.27; range = 26–85) after date of injury (Table 1). The sample included 65 patients who completed a follow-up visit between September 2018 and January 2022. Demographic information and clinical data were extracted from the electronic medical record and included age, sex assigned at birth, race/ethnicity, date of birth, date of hospitalization, Glasgow Coma Scale (GCS) at admission, insurance (public or commercial/private), time elapsed post-injury, neuroimaging findings, mechanism of injury, previous diagnoses of neurodevelopmental conditions, Injury Severity Score (ISS) values, and date of hospital discharge. Patients presenting to the PCCNRP clinic were included in the study if they had been seen within 3 months post-discharge and had complete caregiver-reported and performance-based measures. Participants were excluded if they met criteria for a reading disability preceding injury. Further information regarding PCCNRP policies and procedures have been previously described.^47,48^ The institutional review board at Oregon Health & Science University approved the study procedures with a waiver of consent.

**Table 1.** Demographic Characteristic of Study Participants

Variables	*N* = 65 (%)
Sex	
Male	38 (58.5)
Female	27 (41.5)
Age at admission, years	
*M* (SD)	13.35 (2.65)
Min–Max	8–19
Race	
American Indian/Alaska Native	1 (1.5)
Asian	1 (1.5)
Hawaiian Native/Pacific Islander	2 (3.1)
White	48 (73.8)
More than one race	2 (3.1)
Unknown or not reported	11 (16.9)
Ethnicity	
Hispanic or Latinx	5 (7.7)
Not Hispanic or Latinx	46 (70.8)
Unknown or not reported	14 (21.5)
Insurance	
Private	39 (60)
Public	26 (40)
Distance from home to clinic	
Within 50 miles	27 (41.5)
Further than 50 miles	38 (58.5)
GCS at admission	
*M* (SD)	13.66 (2.88)
Min–Max	3–15
ISS	
*M* (SD)	13.24 (9.38)
Min–Max	1–38
Abbreviated Injury Scale, by region, *M* (SD)	
Head/neck	2.69 (1.36)
Face	1.79 (0.43)
Chest	2.86 (1.03)
Abdomen/pelvis	2.89 (0.93)
Extremities	2.38 (0.72)
External	1.04 (0.19)
Length of hospitalization, days	
*M* (SD)	3.65 (4.61)
Min–Max	1–33
Mechanism of injury	
Motor vehicle accident	18 (27.7)
ATV accident	8 (12.3)
Bike/skateboard/scooter	13 (20)
Pedestrian accidents	9 (13.8)
Fall	10 (15.4)
Other blunt trauma	7 (10.8)
Injury types	
Subdural	12 (28.6)
Epidural	9 (21.4)
Subarachnoid	14 (33.3)
Contusion	15 (35.7)
Mixed or multiple	10 (23.8)
Indeterminate	3 (7.1)
No. of injury types	
*M* (SD)	0.68 (0.87)
Min–Max	0–3
0	34 (52.3)
1	17 (26.2)
2	12 (18.5)
3	2 (3.1)
Injury location	
Frontal	14 (21.5)
Parietal	10 (15.4)
Temporal	11 (16.9)
Occipital	6 (9.2)
Cerebellum	2 (3.0)
Axonal injury	3 (4.6)
None/concussion only	34 (52.3)
No. of injury locations	
*M* (SD)	0.72 (0.88)
Min–Max	0–3
0	36 (55.4)
1	16 (24.6)
2	11 (16.9)
3	2 (3.1)

GCS, Glasgow Coma Scale; ISS, Injury Severity Score; SD, standard deviation; *M*, mean; ATV, all-terrain vehicle.

### Measures

TBI severity was measured and defined by the GCS (pediatric version) recorded at the time of injury (mild 13–15, moderate 9–12, and severe 3–8) and ISS.^49–51^ ISS scores were assigned by trained trauma program staff in accordance with National Trauma Data Bank standards.^52,53^ ISS and GCS scores were utilized in principal analyses. Notably, GCS scores given at admission are known to have multiple confounders, limiting its utility in predicting trajectory and outcomes in pediatric TBI, although research has also highlighted that GCS produced the strongest associations with more severe injuries.^1,54–57^ Additional patient pre-morbid medical or neurodevelopmental disorders were documented and collected from inpatient and clinic notes.

The PCCNRP clinic was utilized to determine the presence of cognitive impairment through implementation of a fixed neuropsychological screening battery consisting of multiple performance-based measures (Table 2).^41^ The Wide-Range Achievement Test (WRAT), 4th Edition (WRAT-4) was given until April 2021, then replaced with the 5th Edition (WRAT-5) to assess Word Reading and provide a proxy, age-adjusted estimate of pre-morbid cognitive functioning.^58–61^ Youth completed either the Children's Memory Scale (CMS) Numbers subtest or the Wechsler Adult Intelligence Scale, Fourth Edition (WAIS-IV) Digit Span subtests to capture attention and working memory performance.^62,63^ The Child and Adolescent Memory Profile (ChAMP) Lists subtest was given to gauge verbal learning and general memory ability.^64^

**Table 2.** Measures Administered by Age at Initial Follow-up Visit

	Age range (years:months)		
	8:0–12:11	13:0–6:11	17:0–19:11
Estimate of pre-morbid functioning			
WRAT-4 or WRAT-5 Word Reading	X	X	X
Attention/working memory			
CMS Digits (forward, backward, and total)	X	X	
WAIS-IV Digit Span			X
Memory & Learning			
ChAMP Lists (immediate and delayed)	X	X	X
Language			
D-KEFS Verbal Fluency (letter and category)	X	X	X
Processing speed			
D-KEFS Trails 2/Number Sequencing	X	X	X
WISC-V Coding and Symbol Search	X	X	
WAIS-IV Coding and Symbol Search			X
Cognitive flexibility			
D-KEFS Trails 4/Number-Letter Switching	X	X	X
Parent/caregiver			
PedsQL (Health Quality of Life Total Score)	X	X	X
BRIEF-2 (Executive Functioning)	X	X	X

WRAT, Wide-Range Achievement Test; CMS, Children's Memory Scale; WAIS, Wechsler Adult Intelligence Scale; ChAMP, Child and Adolescent Memory Profile; D-KEFS, Delis-Kaplan Executive Function System; WISC, Wechsler Intelligence Scale for Children; PedsQL, Pediatric Quality of Life Inventory; BRIEF-2, Behavior Rating Inventory of Executive Functioning, Second Edition.

All patients completed parts of the Delis-Kaplan Executive Function System (D-KEFS) producing scaled scores for Verbal Fluency and Trail Making tasks.^65^ The Verbal Fluency task consisted of a patient's ability to use language and abide by inhibition rules to generate a word based on a single letter or semantic category. For Trail Making, examinees were tasked with connecting and sequencing numbers in order (Condition 2; Number Sequencing) capturing processing speed, whereas the numbers and letters switching task assessed cognitive flexibility (Condition 4; Letter-Number Sequencing).^65^ The Wechsler Intelligence Scale for Children, Fifth Edition (WISC-V) or the WAIS-IV Coding and Symbol Search subtests were administered to assess processing speed.^63,66^

Information regarding daily executive skills was collected by the BRIEF-2 as the parent-report measure.^21^ The BRIEF-2 also provides three additional index scores to evaluate specific areas related to EF: Emotion Regulation Index (ERI), Cognitive Regulation Index (CRI), and Behavior Regulation Index (BRI). The Pediatric Quality of Life Inventory (PedsQL) Parent Related Health Quality of Life (HRQL) was used to collect information regarding caregiver functioning after hospital discharge.^67,68^

### Statistical analysis

All data analyses were completed using SPSS software (Version 27.0; SPSS, Inc., Chicago, IL).^69^ First, to compare differences between patients with and without pre-injury neurodevelopmental conditions, independent samples *t*-tests were used.

Given the high correlations across performance-based EF measures (Supplementary Table S2), principal components analysis (PCA) was conducted to create a single, cumulative neurocognitive index (NCI), thereby reducing the likelihood of type 1 error (Table 3).^43,44,46,47,70^ The maximum number of convergences was set to 25. All eight neurocognitive measures were retained. The WRAT Word Reading subtest was given to provide a proxy, age-adjusted estimate for pre-morbid cognitive functioning.

**Table 3.** Details of Principal Components Analysis

Measures	PCA loading	Inclusion
Combined WISC-V/WAIS-IV Coding	0.75	Retained
Combined WISC-V/WAIS-IV Symbol Search	0.72	Retained
Combined WAIS-IV Digit Span/CMS numbers	0.59	Retained
D-KEFS Trails 4 Switching	0.82	Retained
D-KEFS Phonemic Verbal Fluency	0.70	Retained
D-KEFS Category Fluency	0.76	Retained
ChAMP Lists Immediate	0.64	Retained
ChAMP Lists Delayed	0.72	Retained

Principal components analysis performed on *N* = 65 patients with complete data in all variables. Combining all participant data yielded a single component solution accounting for 51.7% of the total explained variance. Reasons for missing data in cognitive assessments included complications such as orthopedic injury, vision impairment, and behavioral cooperation precluding the completion of some tasks; also, the need for virtual-only visits for some patients assessed during the COVID-19 pandemic.

CMS, Children's Memory Scale; WAIS, Wechsler Adult Intelligence Scale; ChAMP, Child and Adolescent Memory Profile; D-KEFS, Delis-Kaplan Executive Function System; WISC, Wechsler Intelligence Scale for Children; PedsQL, Pediatric Quality of Life Inventory.

Higher NCI scores are reflective of stronger neurocognitive ability. Combining all participant data yielded a single component solution accounting for 51.27% of the total explained variance (Table 3). The NCI included data from the 65 patients who had all eight neurocognitive measures included within the composite, and NCI Z-scores ranged from −2.27 to 2.16. In order to control for estimated pre-morbid cognitive functioning on the NCI, we constructed residualized change scores using a simple linear regression model in which the WRAT Word Reading score predicted NCI.^71,72^ Using this method, more negative residuals represent worse cognitive functioning at follow-up than expected based on estimated pre-morbid functioning, whereas more positive residuals represent better functioning.

In order to explore the relationship between caregiver-reported and performance-based measures of EF, bivariate correlations were then conducted assessing the relationships between the residual of Word Reading and NCI and the BRIEF-2 Global Executive Composite (GEC) score (see Supplementary Table S3 for correlations between performance-based measures of EF and the BRIEF-2 index scores). We also evaluated the relationship between parental impact through caregiver responses on the PedsQL compared to BRIEF-2. Hospital-appointed measures, including the GCS and ISS, were compared to NCI and BRIEF-2. Non-parametric tests were used for variables that did not demonstrate normality with the Kolmogorov-Smirnov goodness-of-fit test.

## Results

Among the 65 patients who completed the follow-up visit, we evaluated 38 males and 27 females 8–19 years of age assessed ∼1–3 months after critical care hospitalization for TBI (Table 1). Most patients were injured by motor vehicle accidents (27.7%), with the remaining categorized by all-terrain vehicle (ATV; 12.3%), biker/skateboard/scooter (20%), pedestrian accident (13.8%), fall (15.4%), and other blunt trauma (10.8%). TBI severity ranged from mild TBI/concussion to severe TBI. GCS at admission was 3–15 (*M =* 13.66, *SD* = 2.88) with ISS scores being 1–38 (*M =* 13.24, *SD* = 9.38) and length of stay 1–33 days (*M =* 3.65, *SD* = 4.61).

### Correlations Behavior Rating Inventory of Executive Functioning, Second Edition, neurocognitive index, and indicators

The following results referring to performance-based neurocognitive outcomes account for the NCI score and estimated pre-morbid functioning with residualized change scores, as described above. Results are similar when original NCI scores are used (see Supplementary Table S1).

Bivariate correlations showed non-significant relationships between NCI and BRIEF-2 GEC and index scores (Table 4). Similarly, the BRIEF-2 GEC score did not significantly correlate with NCI (*r*_(64)_ = −0.02, *p* = 0.10) or injury severity. However, a strong negative correlation was observed between BRIEF-2 GEC and HRQL total score.

**Table 4.** Bivariate Correlation of BRIEF-2 and Neurocognitive Index (Controlling for Word Reading Score)

	GEC	BRI	ERI	CRI
NCI	–0.21	–0.10	–0.21	–0.17

NCI, Neurocognitive Index; GEC, Global Executive Composite; BRI, Behavior Regulation Index; ERI, Emotion Regulation Index; CRI, Cognitive Regulation Index; BRIEF-2, Behavioral Rating Inventory of Executive Functioning, Second Edition.

On the other hand, NCI was strongly, negatively correlated with ISS (Table 5), such that higher severity correlated with worse EF on performance-based measures. Interestingly, NCI did not correlate with GCS at admission or caregiver-rated EF or parent functioning (PedsQL-HRQL). Parent HRQL Summary Score negatively correlated with both injury-severity indicators ISS and GCS at admission (Table 5).

**Table 5.** Bivariate Correlation of Indicators (Controlling for Word Reading Score)

	1	2	3	4	5
1. Neurocognitive Index	1				
2. BRIEF-2 GEC	–0.21	1			
3. Injury Severity Score	–0.35^**^	0.13	1		
4. GCS Admission Mild (13–15) Moderate (9–12) Severe (3–8)	–0.21	0.23	0.25^*^	1	
5. PedsQL Parent Report Total Score	0.08	–0.45^**^	–0.35^**^	–0.32^**^	1

^*^ Correlation is significant at the 0.05 level (two-tailed).

^**^ Correlation is significant at the 0.01 level (two-tailed).

BRIEF-2, Behavior Rating Inventory of Executive Function, Second Edition; GEC, Global Executive Composite; GCS, Glasgow Coma Scale; PedsQL, Pediatric Quality of Life Inventory.

### Pre-injury neurodevelopmental condition

Significant group differences were observed for BRIEF-2 GEC, NCI, Word Reading Score, and the residual of NCI and Word Reading between patients with and without a pre-injury neurodevelopmental condition before admittance to the PICU (Table 6). Those with pre-existing conditions had scores that reflected lower abilities. Because of the small sample size of those with pre-existing conditions (*n* = 14), exploratory correlations were not conducted.

**Table 6.** Independent-Sample *t*-Tests Comparing Participants With and Without History of Developmental Disability

	No pre-injury neurodevelopmental conditions (*N* = 51)		Pre-injury neurodevelopmental conditions (*N* = 14)			
Variable	Mean	SD	Mean	SD	*t* value	*p* value
ISS	12.81	9.81	14.71	7.89	–0.66	0.51
BRIEF-2 GEC	51.96	10.79	62.86	12.91	–3.21	0.002
NCI	0.21	0.93	–0.78	0.88	3.59	<0.001
Word Reading Score	101.08	12.22	92.29	10.42	2.45	0.017
Residual of NCI and Word Reading	0.12	0.69	–0.44	0.99	2.43	0.009
PedsQL Parent Score	77.02	18.91	66.41	19.19	1.84	0.07

ISS, Injury Severity Score; BRIEF-2, Behavior Rating Inventory of Executive Function, Second Edition; GEC, Global Executive Composite; NCI, Neurocognitive Index; PedsQL, Pediatric Quality of Life Inventory.

## Discussion

TBI-related EF impairment, coupled with the effects of family distress post-PICU admission, is an important area of consideration when working with youth in the acute phase of TBI recovery. The present study contributes to the pediatric critical care and TBI literature by providing novel information regarding the relationship between caregiver-reported outcomes, performance-based measures, and hospital-based measures of injury severity in a cohort of pediatric TBI survivors. In support of the study hypotheses, parent-reported outcomes on the BRIEF-2 were not associated with performance-based neuropsychological measures. Results indicate that performance-based assessment and the BRIEF-2 capture different constructs associated with EF, which is consistent with previous research suggesting that the two methods capture different components of EF.^32,33^

The authors of the BRIEF-2 sought to create a measure that describes the day-to-day components and presentations of EF through parent proxy report evaluating a child's ability to modulate behavior, emotions, and metacognitive skills.^21^ Some researchers have suggested that the behaviors rated by parents on this measure may be more related to emotional and behavioral skills (i.e., hot EF), rather than standardized cognition-based neuropsychological assessments administered in a quiet, calm, and distraction-free setting (i.e., cool EF).^37,38,73–76^ As such, caregiver ratings likely reflect hot EF challenges, such as emotional and behavioral dysregulation, which are observed in daily life. By this metric, greater child-reported EF disturbance may reflect higher family burden.^77,78^ In the current study, we found that caregiver-rated EF was strongly correlated with parent-reported distress.

It is also possible that elevated family distress hinders child recovery, though this cannot be determined by this study given that parent ratings of their own distress and their child's EF were given at the same time. Still, this study's findings are consistent with previous research identifying family distress to impact recovery trajectories.^74,79,80^ For instance, clinically elevated levels of family distress have been shown to be a barrier to adherence to clinical recommendations.^81,82^ In addition, parent distress may interact with recovery by reducing parental resources to alleviate and manage children's emotional, cognitive, and physical symptoms.^80–85^

It is also important to consider that some families may under-report symptoms on rating forms both about their own level of distress and about functioning in their children. Research demonstrates that ratings of a child's behavior are likely to be biased because of respondents' perceptions, which are often influenced by contextual factors such as demographic background, societal expectations, and skewed recollections of pre-morbid functioning.^37–39,81^ In these cases, had performance-based measures of EF not been administered, these children's needs would have gone unidentified and untreated. Therefore, a multi-system approach is warranted to effectively identify the culmination of sequalae and family stress observed in this population.^79,84–88^

As expected, there was a significant relationship between expert-rated injury severity and performance-based measures of EF. This relationship remained significant even when accounting for pre-morbid individual differences in cognition indexed by word reading scores, a proxy measure known to remain intact after neurological insult.^61,62,89–92^ Past work shows that EF is vulnerable post-TBI, with these impairments being present early in the acute recovery phase and persisting long term in youth who presented with complicated mild, moderate, or severe TBI and also experienced hospitalization, critical care, and comorbid orthopedic injury.^9,25,28,30,39,41,43,74,86,87^ EF deficits can hinder academic achievement and adaptive functioning in daily life.^90,92^ The current work emphasizes the importance of evaluating for impairment in EF post-TBI, especially in those with greater overall injury severity. Ideally, these children will benefit from neuropsychological evaluations that include both performance-based measures and informant-reported measures to quantify EF. This approach identifies both the impact of TBI on cognitive skills and how these impairments affect daily functioning.

In addition to performance-based and informant-reported measures of EF when considering TBI outcomes, it is also necessary to consider pre-injury abilities. Though the design of our study did not allow for direct measurement of baseline EF pre-injury, we examined the impact of previously identified neurodevelopmental conditions. The past TBI literature has identified lower pre-injury abilities to be a significant risk factor for recovery.^92–95^ Secondary exploratory analyses in the present study revealed differences in EF based on pre-morbid developmental diagnoses, suggesting that pre-morbid conditions may exacerbate or result in diminished EF capacities on both parent report and performance-based measures after TBI. Given our small sample size and inability to directly measure pre-morbid functioning, future research is warranted to further elucidate the connection between pre-injury abilities and EF recovery.

Together, the results demonstrate the differences captured by performance-based and parent reported measures of EF, and also highlight the importance of considering other morbidities, particularly family distress related to PICU admission. We assert that both types of EF measures have merit and add useful information to research and clinical care; however, consistent with previous research, performance-based and informant-report measures appear to capture different constructs related to EF, and utilization of either of these measures in isolation may inaccurately capture EF outcomes, particularly if not accounting for the impact of family distress in an acute pediatric TBI population.^38,39,96^ Overall, the results underscore the need for researchers and professionals to carefully consider the type of information that can be gleaned from various measurement tools, especially when interpreting outcomes to inform treatment and guide intervention.

### Limitations

The present study has several limitations. First, this study is a single-institution, retrospective cohort study with a relatively small sample size largely attributable to the unique population of interest. Additionally, patient TBI severity ranged from mild without intracranial injury to severe with multiple intracranial injuries. Yet, heterogeneity is the rule in community samples drawn from patients admitted and treated in the PICU. Further, there are known geographical and institutional differences regarding acute treatment and critical care approaches, which may limit the generalizability of our findings. Relatedly, broad inferences beyond the scope of this pediatric TBI population are limited. This study was a cross-sectional analysis, thus limiting determination of causation. Further, longitudinal studies are needed too given that all outcome measures were collected 1–3 months after hospital discharge. Future research with greater statistical power is needed to explore the contribution of injury characteristics on outcomes.

## Conclusion

The present study revealed a non-significant association between caregiver-reported measures and performance-based measures of EF in the acute-recovery period after pediatric TBI. The findings demonstrate the importance of multi-method assessment to detect potential acquired deficits to inform rehabilitation. Further, these findings are consistent with previous literature attesting that performance-based measures may underestimate behavioral and emotional impairments associated with EF.^47,76,81^ Overall, these findings evince that the BRIEF-2 and neuropsychological assessment measure different constructs, with the BRIEF-2 capturing subtle behavioral problems, parental psychological distress, and emotional constructs associated with pediatric TBI recovery. Conversely, performance measures of EF may capture specific functional neurocognitive deficits directly related to injury severity. Moreover, our study demonstrates that BRIEF-2 scores are related to measures of caregiver distress, and therefore using this measure as a primary clinical outcome for cognition requires the caveat that it also reflects other morbidities related to PICU admission.

## Supplementary Material

Supplemental data

Supplemental data

Supplemental data

## Abbreviations Used

BRI: Behavior Regulation Index
BRIEF-2: Behavior Rating Inventory of Executive Functioning, Second Edition
ChAMP: Child and Adolescent Memory Profile
CMS: Children's Memory Scale
CRI: Cognitive Regulation Index
D-KEFS: Delis-Kaplan Executive Function System
EF: executive functioning
ERI: Emotion Regulation Index
GCS: Glasgow Coma Scale
GEC: Global Executive Composite
HRQL: Health Quality of Life
ISS: Injury Severity Score
*M*: mean
NCI: Neurocognitive Index
PCA: principal component analysis
PCCNRP: Pediatric Critical Care Neurotrauma Recovery Program
PedsQL: Pediatric Quality of Life Inventory
PICU: pediatric intensive care unit
SD: standard deviation
TBI: traumatic brain injury
WAIS: Wechsler Adult Intelligence Scale
WISC: Wechsler Intelligence Scale for Children
WRAT: Wide-Range Achievement Test
