# Supplementary material for: Variability Across Caregiver and Performance-Based Measures of Executive Functioning in an Acute Pediatric Neurocritical Care Population
Source: Neurotrauma Rep. 2023 Mar 1;4(1):97–106. doi: 10.1089/neur.2022.0083 (PMC9989517; doi:10.1089/neur.2022.0083)
Supplement: Supplemental data [file Supp_TableS2.docx]

**Supplemental Table 2**

*Bivariate Correlation Executive Functioning Measures*

| Executive Functioning Measures | | | | | | | | | |
| --- | --- | --- | --- | --- | --- | --- | --- | --- | --- |
|  | 1 | 2 | 3 | 4 | 5 | 6 | 7 |  |  |
| 1. Coding  (WAIS-IV/WISC-V) | 1 |  |  |  |  |  |  |  |  |
| 2.  Symbol Search  (WAIS-IV/WISC-V) | 0.621** | 1 |  |  |  |  |  |  |  |
| 3. Lists Immediate  (ChAMP) | 0.325** | 0.358** | 1 |  |  |  |  |  |  |
| 4. Lists Delayed  (ChAMP) | 0.406** | 0.455** | 0.679** | 1 |  |  |  |  |  |
| 5. Digits Total  (WAIS-IV/CMS) | 0.392** | 0.320** | 0.488** | 0.318** | 1 |  |  |  |  |
| 6. D-KEFS  Letter Fluency | 0.337** | 0.331** | 0.382** | 0.438** | 0.313* | 1 |  |  |  |
| 7. D-KEFS  Category Fluency | 0.576** | 0.429** | 0.409** | 0.414** | 0.302* | 0.592** | 1 |  |  |
| 8. D-KEFS Number Letter Switching | 0.621** | 0.635** | 0.566** | 0.568** | 0.478** | 0.427** | 0.556** | 1 |  |
| 9. Word Reading  (WRAT4/WRAT5) | 0.438** | 0.460** | 0.483** | .483** | 0.439** | 0.374** | 0.578** | 0.578** | 1 |

*Notes:* **Correlation is significant at the 0.01 level (2-tailed) and *Correlation is significant at the 0.05 level (2-tailed). NCI, Neurocognitive Index; GEC, Global Executive Composite; BRI, Behavior Regulation Index; ERI, Emotion Regulation Index; CRI, Cognitive Regulation Index; WAIS, Wechsler Adult Intelligence Scale, Fourth Edition; WISC-V, Wechsler Intelligence Scale for Children, Fifth Edition; CMS, Children’s Memory Scale; ChAMP, Child and Adolescent Memory Profile; D-KEFS, Delis-Kaplan Executive Function System; WRAT4/WRAT5, Wide Range Achievement Test, Fourth Edition or Fifth Edition.
