# Supplementary material for: Variability Across Caregiver and Performance-Based Measures of Executive Functioning in an Acute Pediatric Neurocritical Care Population
Source: Neurotrauma Rep. 2023 Mar 1;4(1):97–106. doi: 10.1089/neur.2022.0083 (PMC9989517; doi:10.1089/neur.2022.0083)
Supplement: Supplemental data [file Supp_TableS3.docx]

**Supplemental Table 3**

*Bivariate Correlation Between BRIEF-2 and Measures of Executive Functioning*

|  | GEC | BRI | ERI | CRI |
| --- | --- | --- | --- | --- |
| Coding  (WAIS-IV/WISC-V) | -0.307* | -0.195 | -0.220 | -0.263* |
| Symbol Search  (WAIS-IV/WISC-V) | -0.108 | -0.038 | -0.027 | -0.052 |
| Lists Immediate  (ChAMP) | -0.048 | -0.107 | -0.161 | -0.077 |
| Lists Delayed   (ChAMP) | -0.217 | -0.189 | -0.225 | -0.164 |
| Digits Total  (WAIS-IV/CMS) | -0.064 | -0.001 | -0.004 | -0.047 |
| D-KEFS   Letter Fluency | -0.079 | -0.039 | -0.002 | 0.007 |
| D-KEFS  Category Fluency | -0.302* | -0.225 | -0.272* | -0.226 |
| D-KEFS Number Letter Switching | -0.245* | -0.150 | -0.193 | -0.171 |
| Word Reading  (WRAT4/WRAT5) | -0.142 | -0.147 | -0.048 | -0.046 |

*Notes:* **Correlation is significant at the 0.01 level (2-tailed) and *Correlation is significant at the 0.05 level (2-tailed); BRIEF-2, Behavior Rating Inventory of Executive Function, Second Edition; GEC, Global Executive Composite; BRI, Behavior Regulation Index; ERI, Emotion Regulation Index; CRI, Cognitive Regulation Index; WAIS, Wechsler Adult Intelligence Scale, Fourth Edition; WISC-V, Wechsler Intelligence Scale for Children, Fifth Edition; CMS, Children’s Memory Scale; ChAMP, Child and Adolescent Memory Profile; D-KEFS, Delis-Kaplan Executive Function System; WRAT4/WRAT5, Wide Range Achievement Test, Fourth Edition or Fifth Edition.
