# Supplementary material for: Variability Across Caregiver and Performance-Based Measures of Executive Functioning in an Acute Pediatric Neurocritical Care Population
Source: Neurotrauma Rep. 2023 Mar 1;4(1):97–106. doi: 10.1089/neur.2022.0083 (PMC9989517; doi:10.1089/neur.2022.0083)
Supplement: Supplemental data [file Supp_TableS1.docx]

**Supplemental Table 1**

*Bivariate Correlation of Indicators (not controlling for Word Reading Score)*

|  | 1 | 2 | 3 | 4 | 5 |
| --- | --- | --- | --- | --- | --- |
| 1. Neurocognitive Index (NCI) Residual and Word Reading Score | 1 |  |  |  |  |
| 2. BRIEF-2 GEC | -.21 | 1 |  |  |  |
| 3. Injury Severity Score (ISS) | -.30** | .13 | 1 |  |  |
| 4. GCS Admission  Mild (13-15)  Moderate (9-12)  Severe (3-8) | -.11 | .23 | .25* | 1 |  |
| 5. PedsQL Parent Report Total Score | .26 | -.45** | -.35** | -.32** | 1 |

*Notes:* **Correlation is significant at the 0.01 level (2-tailed) and *Correlation is significant at the 0.05 level (2-tailed). BRIEF-2, Behavior Rating Inventory of Executive Function, Second Edition; GEC, Global Executive Composite; GCS, Glasgow Coma Scale; PedsQL, Pediatric Quality of Life inventory.
